# Platelet indices in children with type 1 diabetes mellitus: a simple glucoregulation monitoring tool

**DOI:** 10.4314/ahs.v23i4.35

**Published:** 2023-12

**Authors:** Dejan Dobrijević, Jelena Antić, Goran Rakić, Ljiljana Andrijević

**Affiliations:** University of Novi Sad, Faculty of Medicine, Novi Sad, Serbia; Institute for Child and Youth Health Care of Vojvodina, Novi Sad, Serbia

**Keywords:** Diabetes mellitus, children, platelet, laboratory diagnosis, control group, inflammation

## Abstract

**Introduction:**

Long-term hyperglycemia can lead to changes in the function and morphology of platelets.

**Objective:**

This study aimed to test the potential glucoregulation monitoring properties of platelet indices, mean platelet volume (MPV) and platelet distribution width (PDW), in children with type 1 diabetes mellitus (T1DM).

**Methods:**

The study included 453 patients below the age of 18 with T1DM treated at the Institute for Child and Youth Health Care of Vojvodina. Children were divided into two groups, according to their glucoregulation quality, i.e., glycated hemoglobin (HbA1c) levels. Descriptive and inferential statistical analyses were performed.

**Results:**

MPV and PDW were found to be important in predicting poor glucoregulation, both in independent and conjoint analysis. Proposed cut-off values for MPV and PDW in the glucose control monitoring of children with T1DM were 7.6 fL and 14.4%, respectively.

**Conclusion:**

Our study showed that MPV and PDW have monitoring properties in terms of glucose control in children with T1DM. Additionally, our study emphasizes the importance of selecting the most convenient control group in order to avoid misleading conclusions.

## Introduction

Type 1 diabetes mellitus (T1DM) is the most common metabolic disease characterized by disturbances in carbohydrate, fat and protein metabolism. It is caused by a chronic lack of insulin due to genetically determined destruction of pancreatic beta cells. It has a great socio-medical importance considering its common complications, morbidity and mortality.^1^ More than 18,000 children are newly diagnosed with T1DM annually.^2^

The main laboratory characteristic of this disease is an elevated level of plasma glucose. Long-term hyperglycemia leads to covalent binding of glucose to platelet membrane proteins. Insulin inhibits this process, and in the case of its deficiency, this reaction rate increases.^3^

When such platelets are passed through the channel of a flow cytometer on a hematology analyzer, they will be larger in volume than they actually are. The main laboratory indicators of platelet size are mean platelet volume (MPV) and platelet distribution width (PDW). Therefore, these parameters could theoretically have diagnostic and prognostic properties in terms of glucoregulation.^3^,^4^ According to American Diabetes Association (ADA), achieving glycated hemoglobin (HbA1c) targets of < 7.0% is considered a good glycemic control in patients with T1DM.^5^

Recent studies found that MPV was associated with inflammatory conditions such as type 2 diabetes mellitus^6^, diabetic nephropathy^7^, hypothyroidism^8^, vertebral discopathies^9^, irritable bowel disease^10^ etc. On the other hand, PDW has been introduced as a predictor of underlying inflammation in diabetic nephropathy^11^, infections^12^, cardiac diseases^13^, autoimmune liver diseases^14^, and other gastrointestinal conditions^15^.

There are numerous studies about the utility of platelet indices in people with diabetes mellitus, but they were mostly conducted in the adult population.^16^ Data on the utility of platelet indices in children with T1DM are still limited. In addition, existing studies are mostly designed to compare children with T1DM to a control group of healthy children while testing only diagnostic and not monitoring properties of platelet indices.^17^ Additional research is needed to confirm potential monitoring properties of platelet indices in pediatric diabetology.

## Objective

This study aimed to test the potential glucoregulation monitoring properties of platelet indices (MPV and PDW) in children with T1DM.

## Methods

This single-center, cross-sectional study included children under the age of 18 with diagnosis of T1DM without complications (ICD-10 code: E10.9), who were examined in the Endocrinology department at the Institute for Children and Youth Healthcare of Vojvodina in Novi Sad, Serbia, between January 2019 and October 2022. In that period, in total 558 children with T1DM were examined. Following the exclusion criteria, in total 453 individuals were selected for the study. Exclusion criteria were: acute and chronic diabetic complications, hematological diseases, cancer, chronic diseases, usage of medication that could affect platelet count and missing data. Children were divided into two groups, according to ADA glucoregulation criterion of HbA1c<7.0%.

Pletelets (TIT), plateletcrit (PCT), MPV and PDW values on the day of admission were recorded using the Institute's laboratory information system data. The reference interval of PLT is 150 – 400 x 109/L, PCT is 0.12 – 0.36%, MPV is 6.0 – 13.0 fL and in the case of PDW it is 10.0 – 17.0%. The values were tested on hematology analyzer Advia 2120 (Siemens Healthcare, Erlangen, Germany).

Statistical analyses (descriptive and inferential) were performed using the Statistical Package for the Social Sciences (SPSS version 26.0) software (IBM Corporation, Armonk, New York, United States). Significance level for calculated differences was set at 0.05. A Chi-square test was employed to determine the differences between nominal variables. Normality of distribution for continuous variables was tested via Shapiro–Wilk test. Differences between the groups were analysed using the Mann-Whitney U test. Univariate logistic regression analysis was employed to test if the parameters could predict poor glucose control. Discrimination between groups was estimated by performing the Receiver operating characteristics (ROC) analysis.

## Results

From January 2019 and October 2022 (Table 1), 558 children with T1DM were examined at our Institute. Following the exclusion criteria, in total 453 cases were selected for the study, 244 girls and 209 boys with the median age of 14 years. According to ADA glucoregulation criterion of HbA1c<7.0%, 264 children had good and 189 poor glucose control. The female share of the first group (HbA1c<7.0%) was 44.7% with the median age of 13.5 years. The female share of the second group (HbA1c>7.0%) was 48.1% with the median age of 14 years.

**Table 1:** Demographic and laboratory data within groups

	HbA1c < 7.0% (n=264)	HbA1c > 7.0% (n=189)		p-value
**female/male (n)** ^**#**^	118/146	91/98		0.467
**age (y)** ^**†**^	13.5 (10 – 17)	14 (11 – 16)		0.329
**PLT (10^9^/L)**	287 (208.2 – 380.2)	309 (231 – 379.5)		0.253
**PCT (%)** ^**†**^	0.22 (0.17 – 0.28)	0.24 (0.17 – 0.31)		0.131
**MPV (fL)** ^**†**^	7.6 (6.8 – 8.1)	7.9 (7.4 – 8.4)		**<0.001**
**PDW (%)** ^**†**^	14.4 (13.7 – 16.2)	15.7 (12.9 – 17)	15.1 (13.1 – 16.3)	**0.033**

# Values are numbers; Chi-square test.

† Values are median (interquartile range: Q1–Q3); Mann-Whitney U test.

PLT – Platelets. PCT – Plateletcrit. MPV - Mean platelet volume. PDW - Platelet distribution width. Values in bold are statistically significant.

Significant differences between the groups regarding sex and age were not observed (p>0.05). Binary logistic regression analysis showed that age was not a risk factor for poor glucose control (p = 0.073; OR: 1.050; 95% CI: 0.995 – 1.108). Significant differences between the groups were observed in MPV (p<0.001) and PDW values (p=0.033), while the differences regarding PLT and PCT were not significant (Table 1).

Employing logistic regression analysis, MPV and PDW were found to be important independent predictors (Table 2).

**Table 2:** Univariate regression analysis between groups

	p-value	Odds ratio	95% CI	

			Lower	Upper
**MPV (fL)**	<0.001	2.200	1.179	2.816

**PDW (%)**	0.04	1.080	1.004	1.162

MPV - Mean platelet volume. PDW - Platelet distribution width.

**Figure 1:**
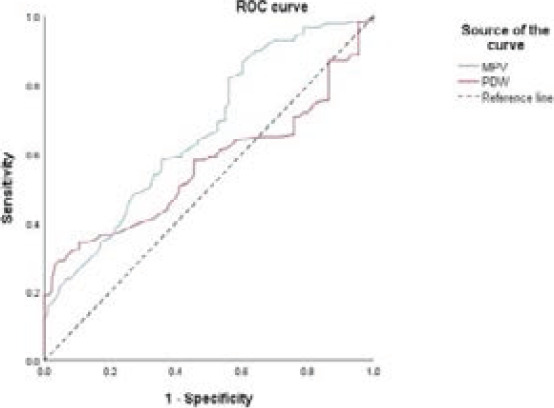
Receiver operating characteristic curve for MPV and PDW in glucoregulation monitoring of type 1 diabetes mellitus in children (ROC - Receiver operating characteristic. MPV - Mean platelet volume. PDW - Platelet distribution width.)

Receiver operating characteristics analysis was employed to assess the glucose control monitoring properties of MPV and PDW regarding sensitivity and specificity (Figure 1).

Proposed cut-off values for MPV and PDW in the glucose control monitoring of children with T1DM were 7.6 fL and 14.4%, respectively (Table 3). The parameters showed better sensitivity than specificity.

**Table 3:** Proposed cut-off values for platelet indices in monitoring of type 1 diabetes mellitus in children

	AUC	Std. Error	95% CI	Cut-Off Value	Sensitivity %	Specificity %
**MPV (fL)**	0.666	0.025	0.617 – 0.716	7.6	64.0	53.4
**PDW (%)**	0.564	0.029	0.507 – 0.621	14.4	58.7	51.1

AUC - Area under the curve. MPV - Mean platelet volume. PDW - Platelet distribution width.

## Discussion

Diabetes management includes healthy eating, regular exercise and plasma glucose monitoring.^18^ In addition to the quantification of blood plasma glucose, the most common indicator of the glucoregulation qualiy is HbA1c. According to ADA, the target value of HbA1c for people with T1DM is less than 7.0%.5 The need for additional, simpler and cheaper biomarkers is a constant goal of laboratory medicine.

Platelet indices, unlike other diagnostic modalities that can indicate changes in the body caused by hyperglycemia, represent an easily accessible, inexpensive and technically undemanding tool. Most hematology counters measure these parameters as part of a complete blood count.^19^

MPV represents both the volume of platelets and the measure of their functionality. Macroplatelets (high MPV) are present when platelet formation is reduced. However, in the state of prolonged hyperglycemia, platelet membrane proteins are being glycated, which is why they become larger.^20^ Another reason why the volume of platelets in T1DM increases is the water accumulation in their cytosol in hyperglycemic states. Therefore, it is important for the clinician to understand these biochemical mechanisms in order to interpret high MPV values properly.^21^ In our study, we confirmed a presence of statistically significantly larger platelets in the blood of patients with poorly regulated T1DM compared to patients who had good glucose control. Zaccardi et al.^22^ confirmed that a long-term glucose control influenced platelet mass and the volume-count relationship in T1DM subjects. In another study conducted by Söbü et al.,^23^ it has been noted that as the duration of diabetes and HbA1c levels increased, the MPV levels also increases.

PDW is a measure of heterogeneity in the morphology of platelets, i.e., it indicates how much platelets differ from each other in size.^24^ Additionally, it is an indicator of their prothrombotic and proinflammatory function. Therefore, this parameter can also be of importance both for the assessment of glucoregulation and risk of micro- and macrovascular complications in hyperglycemic states.^25^ In our study, significantly higher values of this index were confirmed in the blood of patients with poorly regulated T1DM compared to patients who had good glucose control. In the study by Venkatesh et al.,^26^ PDW values were significantly higher in children with suboptimal glycemic control compared to optimal control. Khudhur et al.^27^ reported that PDW was significantly higher among children with T1DM in comparison with the control group.

On the other hand, there is some data discrepancy in the literature. Namely, in the study about platelet parameters in 80 children with T1DM, Korkmaz^28^ reported no significant difference in MPV and PDW values between patients and controls. In this study, the control group consisted of healthy children. We believe that this is the reason for not recognizing the importance of this parameter, because pathophysiologically, there should be no differences between healthy children and children with good glucose regulation.

Platelet indices have potential for clinical utility as a simple glucoregulation monitoring tool in children with T1DM, supported by above-mentioned authors' evidence and similar clinical laboratory studies. Monitoring properties of MPV and PDW could help pediatricians in more effective follow-up of children with T1DM.

This study has some limitations. Only a limited number of children could be evaluated since this was a single-center study. Additionally, the data were recorded using the Institute's laboratory information system. With more detailed analysis about some clinical features, e.g., disease duration, we could create subgroups and draw more precise and potentially different conclusions.

## Conclusion

Our study showed that MPV and PDW have monitoring properties in terms of glucose control in children with T1DM. In perspective, these parameters could be implemented in the various classifying algorithms in order to achieve a better insight into T1DM management quality and subsequently to apply the best treatment on time. Additionally, with our study we emphasize the importance of selecting the most convenient control group in order to avoid misleading conclusions.
